# Use of a cow-side oestrus detection test for fertility management in Kenyan smallholder dairy herds.

**DOI:** 10.12688/gatesopenres.13542.2

**Published:** 2022-09-20

**Authors:** Andrew R. Peters, Johanna T. Wong, Erin J. Williams, Bridgit S. Muasa, Nathaniel F. Makoni, Chris M. Ngige, Fiona K. Allan, Michael Christian, Peter J.H. Ball

**Affiliations:** 1 Centre for Supporting Evidence Based Interventions-Livestock, Royal (Dick) School of Veterinary Studies, University of Edinburgh, Edinburgh, EH25 9RG, UK; 2 Royal (Dick) School of Veterinary Studies, University of Edinburgh, Edinburgh, EH25 9RG, UK; 3 African Breeders Services Total Cattle Management Ltd (ABS TCM Ltd), Nairobi, Kenya

**Keywords:** Dairy cattle, smallholder, artificial insemination, progesterone, oestrus detection

## Abstract

**Background: **The use of artificial insemination (AI) has great potential to improve smallholder dairy herds in Africa, however poor success and, in some situations, high costs in Kenya, have been discouraging.  Effective AI requires accurate oestrus detection and the measurement of progesterone (P4) can be used to indicate oestrus as well as non-pregnancy.  A cow-side progesterone lateral flow test, *P4 Rapid*, was evaluated as an aid to detect oestrus and non-pregnancy in Kenyan dairy cows, and assessed for association with AI efficiency.

**Methods: **A total of 527 cows were enrolled in the study, from two counties in central and southern Kenya.  Cattle in the test group (n = 308) were presented when suspected to be in oestrus and tested with the *P4 Rapid* (low P4 = oestrus, medium P4 = inconclusive, high P4 = not in oestrus/pregnant).  Cattle with low P4 were inseminated.  Cattle in the control group (n = 219) were inseminated when oestrus behaviour was detected i.e. standard practice.

**Results: **Of the total *P4 Rapid* tests performed (n = 745), 1.5% were inconclusive, with the true accuracy of the test between 87-97%.  Conception rates were not significantly higher in the test group (83.9%) compared to the control group (77.9%). Abortion rates were not significantly different between the control (9.5%) and test groups (8.2%).  In the test group, 6.2% (19/308) cows showed a medium or high P4 level on day 0 and nine of these were subsequently found to have been already pregnant.

**Conclusions: **The data indicated that the P4 Rapid test can be a useful tool to assist farmer decision-making in the confirmation of correct timing for AI, and importantly may avoid unnecessary inseminations in pregnant animals, thus reducing the risk of AI-induced abortion.

## Introduction

In Kenya, as in many lower- and middle-income countries, the use of artificial insemination (AI) has the potential to improve cattle genetics and reproductive performance in smallholder dairy herds ( Gicheha *et al.*, 2019). Furthermore, improving access to breeding services and thus increased access to quality genetic material would make smallholder dairy farming more sustainable and economically viable ( Murage & Ilatsia, 2011; Odero-Waitituh, 2017). However, the associated high costs, coupled with low success rates, has meant that efforts to improve livestock production via reproductive technologies, including AI, have tended to fail ( Lawrence *et al.*, 2015; Omondi *et al.*, 2017; Rademaker *et al.*, 2016). Evidence suggests that dairy farmers in Kenya are keen to use AI services on their farms ( Khainga *et al.*, 2018; Omondi *et al.*, 2017) but need to be convinced of its efficacy in order for uptake to be optimised ( Murage & Ilatsia, 2011).

A major contributing factor to poor reproductive performance in smallholder dairy farms in Kenya has been poor oestrus detection rates ( Mwai *et al.*, 2020; Owen, 2005), with mistiming of AI leading to poor conception rates and increased calving intervals ( Mungube *et al.*, 2014). Effective AI requires efficient and accurate detection of ovulation, traditionally signalled by observed oestrus behaviour. Oestrus behaviour typically lasts between six and 30 hours and is dependent on cow and seasonal factors, with the main sign of oestrus standing to be mounted by a bull, or other cows ( Ball & Peters, 2004c). Risk factors for poor expression of oestrus can be environmental factors such as nutrition, housing, season and number of herd mates in oestrus simultaneously, or cow factors such as silent or anovulatory oestrus and adverse health conditions ( Roelofs *et al.*, 2010). An inability to easily detect oestrus, coupled with poor expression of behavioural signs of oestrus by the cow can hinder insemination at the correct time ( Walsh *et al.*, 2011). Even when farm staff are experienced, many oestrus events can still be missed, and ‘false’ oestrus, resulting from abnormal cow behaviour or human error, can also occur. Even in the most sophisticated intensive dairy systems in high-income countries, oestrus detection remains an obstacle to optimal reproductive efficiency ( Adenuga *et al.*, 2020). 

Calving to first service interval and first service to conception interval are both dependent on the rate of oestrus detection as well as the herd conception rate ( Esslemont & Ellis, 1974). The early detection of pregnancy is also essential in managing efficient dairy cow reproduction or rather non-pregnancy so that the appropriate management decision can be implemented e.g. repeat insemination ( Fricke *et al.*, 2016). Overall pregnancy rates to AI have been disappointingly low in African countries ( Omondi *et al.*, 2017). In Kenya, the estimated national average insemination rate ranges from 1.5 to three per cow per conception ( Elving *et al.*, 1979; Ministry of Livestock Development, 2010; Mungube *et al.*, 2019; Mutiga *et al.*, 1994; Odima *et al.*, 1994; PICO-Eastern Africa, 2014).

As progesterone (P4) is a hormonal product of the *corpus luteum*, its measurement can be applied as a confirmatory test to the two above situations, i.e. to verify accurate detection of oestrus on the day of insemination, or to confirm non-pregnancy approximately 21 days after insemination by the finding of a low P4 concentration in blood or milk. The measurement of bovine P4 has evolved over the past four to five decades from laboratory based radio-immunoassay through enzyme-linked immunosorbent assay (ELISA) techniques to cow-side lateral flow tests or in-milk-line monitoring ( Samsonova *et al.*, 2018). The cow-side *P4 Rapid* lateral flow test was developed in the UK (Ridgeway Science, UK) and studies comparing its performance to other assay methods have been published ( Ingenhoff *et al.*, 2016; Kočíková *et al.*, 2019; Muasa, 2020; Omontese *et al.*, 2020; Waldmann & Raud, 2016).

The present study was carried out to evaluate the use of the cow-side *P4 Rapid* progesterone lateral flow test in confirmation of oestrus, as well as non-pregnancy in dairy cows in Kenya and to determine whether its application was associated with increased effectiveness of AI.

## Methods

### Study area

**Figure 1.**
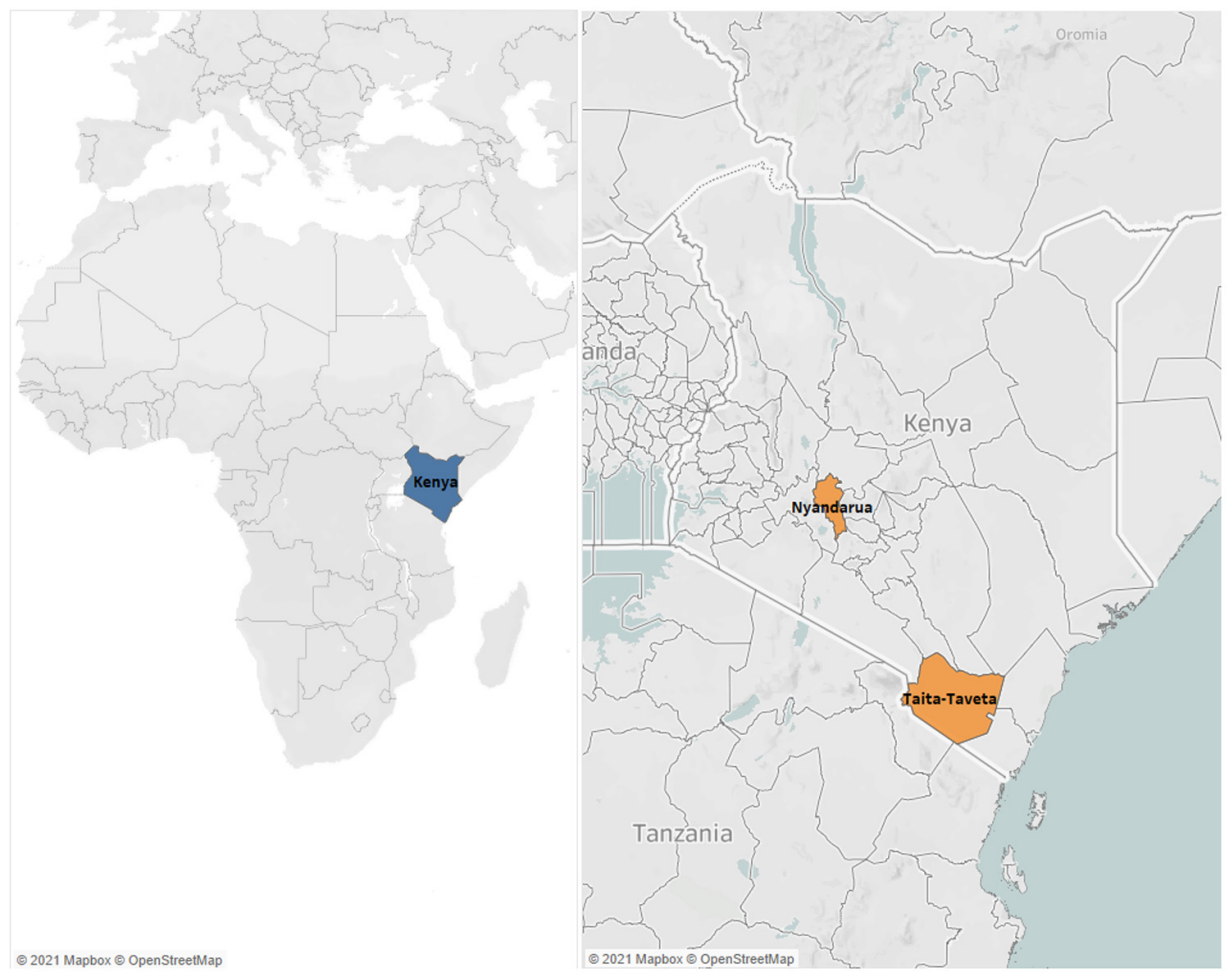
Location of study areas. The map on the left shows the position of Kenya (highlighted in blue) within Africa. The map on the right shows the study areas, Nyandarua and Taita-Taveta (highlighted in orange) within Kenya.

The study was conducted in two counties in Kenya ( Figure 1), between August 2019 and October 2020. The two counties, Nyandarua and Taita-Taveta, were areas where farmers utilise artificial insemination service providers (AISPs). In Nyandarua, farmers typically practice mixed farming – both intensive (zero-grazing) and extensive (free-range), with some farmers practicing semi-zero (zero-grazing in the morning and free-range in the afternoon). Cattle in this area mostly comprise Ayrshire x Friesian crosses, with very few pure breeds. Herd sizes ranged from three to seven cattle. In Taita-Taveta, zero-grazing is widely practised. Cattle breeds in this area were also mostly Ayrshire x Friesian crosses, very few pure breeds, and some indigenous zebu x Ayrshire/Friesian cross breeds, with herd sizes of two or three cattle.

### Participant selection and characteristics

Convenience sampling of farmers was based on those farmers residing in areas near to AISPs (Nyandarua farms n = 220; Taita-Taveta farms n = 177). The AISPs contacted their clients to recruit participants into the study. All healthy, lactating cows eligible for AI on the farms were included unless they were likely to be removed from the farm in the following 90 days. Body condition score and health information were recorded for each cow, including cases of mastitis and other infections and cows were excluded if they were suffering from a health issue which could affect fertility.

Following a voluntary waiting period of 60 days postpartum at the commencement of the breeding season, cows were allocated to either the *P4 Rapid* test group or a control group (no testing). To reduce bias, the first cow on each farm presented for insemination was randomly assigned to either group then subsequent cows were assigned alternately to the control or test group.

### Reproductive management

**Figure 2.**
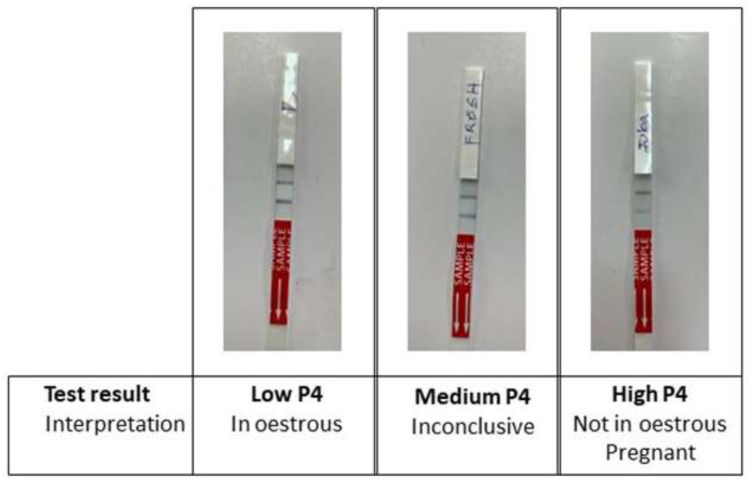
Ridgeway P4 Rapid test results and interpretation. The lower line is the test line (in response to progesterone) and the upper line is the reference/control line (indicates that the test has run correctly). Low P4 is indicated by a stronger test line; medium P4 is indicated by equal lines; high P4 is indicated by a weaker test line.

Oestrus was determined by the farmer by observing behavioural signs and oestrous cycle tracking as described by Ball and Peters ( Ball & Peters, 2004a). When oestrus was observed in cows in the test group, a milk sample was collected that day, usually at the next milking, and progesterone levels tested by the *P4 Rapid* test ( Figure 2).

AI and progesterone measurements were carried out by the AISPs who would normally, in the course of established herd management, inseminate the cows enrolled on the study. Prior to commencement of the study, a detailed training course (see *Extended data;* Allan, 2022) was provided for the AISP technicians to explain the rationale for the study and the theory behind the *P4 Rapid* Test, as well as to demonstrate the test procedure. 

### Consents and ethical approval

Participating farmers gave verbal approval for inclusion in the trial to the AISPs. Verbal rather than written consent was taken as literacy levels in the study audience were low. All data were recorded on Open Data Kit (ODK) and informed consent was documented when the AISP added a new farmer to the software. If permission was not given or the permission script not read, then the ODK refused to record any data. Ethical approval for the trial was sought from University of Edinburgh, Veterinary Ethical Review Committee but was deemed to be out-with requirements as it did not involve animal interventions other than carried out under routine farming processes i.e. milking of cows.

### Study design / protocol

A prospective, semi-experimental and controlled study design was used to select cattle eligible for AI. Sample size was calculated for conception (success or failure). The required total sample size was 346, sufficient for establishing a 15% difference in conception rates between the test and control groups, at 95% confidence and 80% power. Additional cows were recruited on the assumption that there would be a significant number of cows excluded from final analysis due to incomplete data, thus resulting in a total recruitment of 527 cows. 

According to the study protocol, for each farm, cows were alternately allocated to control or test groups. The control group emulated standard farm practice; farmers presented cows to AISP for insemination when the farmer suspected the cow was in oestrus, either from cycle tracking or the cow exhibiting behavioural oestrus. Cows in the control group were artificially inseminated 12 hours after first observed in standing oestrus. Farmers could re-present their cows for a follow-up service if they thought the cow came back into heat at a later date during the study. For the test group, farmers similarly presented their cows when they were thought to be in oestrus. The cows were then tested with the Ridgeway *P4 Rapid* cow-side oestrus detection test. In cycling cows, a low P4 level (≤2 ng/ml) indicates that the cow is in oestrus; a medium P4 level (>2 - ≤3.5 ng/ml) is inconclusive; a high P4 level (>3.5 ng/ml) ( Muasa, 2020) indicates the cow is either not in oestrus or pregnant ( Figure 2).

Test group cows that had low P4 were inseminated. Cows that returned a medium or high P4 test result were not inseminated, and farmers were requested to present these for re-testing when the cow was thought to be on heat again [or at day 21 and day 42]. On repeat visits, farmers were offered the option of AI if the *P4 Rapid* test results were low.

The outcome of the inseminations was evaluated by pregnancy diagnosis (PD) between 60 and 90 days after insemination, either by rectal palpation or ultrasound, or by obtaining calving information.

***P4 Rapid test.*** Ridgeway Science *P4 Rapid* is a lateral flow test for the detection of progesterone (P4) in milk (Ridgeway Science, UK). The test is a paper immune strip embedded with an anti-P4 antibody. A test strip was labelled with the cow identification and data before dipping into approximately 7 ml of milk for testing. A response was observed after approximately 10min, whereby the colour intensity of the test line was inversely proportional to the concentration of progesterone in the milk ( Figure 2). Milk was tested at ambient temperature, within three hours from sample collection, following manufacturer guidelines.

Sensitivity and specificity of the P4 Rapid test had been evaluated previously ( Muasa, 2020; Waldmann & Raud, 2016).

### Data analysis

Data were exported to Microsoft Excel (version 2013), where they were cleaned. To assess test performance and mistimed AI, all enrolled animals were analysed. For outcomes that were compared between test and control groups, namely conception rate, abortion rate and days to conception, a subset of the data were used, where cows that deviated from the study protocol, had incomplete or ambiguous data were removed. Additionally, only cows that conceived at recorded AI dates were retained - those pregnant to unrecorded AI dates may have been inseminated elsewhere or may have had access to a bull. Cows known to be pregnant by the end of the study period (taken as 1 December 2020) were counted as having conceived at their last AI date if they were served on or after 15 February 2020.

Statistical summaries were produced using Excel and R (version 4.0.3) via RStudio (version 1.3.1093). Chi-squared (χ ^2^) analysis was used to compare the differences between groups. Statistical tests were considered significant at P ≤ 0.05.

## Results

A total of 527 cows were enrolled in the study, of which 308 were in the test group and 219 in the control group. The outcomes of all enrolled cattle are presented in the *Underlying data* ( Allan, 2022), showing data retained or removed for analysis. In the data subset, a total of 174 cattle remained in the test group (134 removed) and 149 remained in the control group (70 removed).

### Mis-timed artificial insemination

In the test group, 6.2% (19/308) cows showed a medium or high P4 level on day 0. Of these, nine were already pregnant (six subsequently calved, one aborted, one had a stillbirth, and one was accidentally served on day 0 but confirmed already pregnant on PD at a later date), six were not pregnant and assumed as not being in oestrus (two cows were accidentally inseminated at day 0 – of these, one did not conceive and was not inseminated when subsequently on heat, and one was subsequently diagnosed as not pregnant; two cows subsequently came on heat but were not inseminated; and two were pregnant and calved from subsequent AI), and four cows were lost to follow up.

In the control group, based on calving dates, 2.3% (5/219) cows inseminated on day 0 were already pregnant. It is not known how many control cows that aborted or had stillbirths were pregnant at the time of AI.

Overall, at least 4.6% (24/527) of all enrolled cows could have avoided an unnecessary AI had their P4 levels been known prior to insemination and the study protocol correctly followed.

### Test performance

Within the test group, 206 cows had at least one *P4 Rapid* test result that could be linked with either being in oestrus (cow was served and subsequently conceived), or a known pregnancy status (diagnosed as pregnant or non-pregnant or was pregnant and aborted or had a stillbirth). These *P4 Rapid* test results were consequently submitted for data analysis.

Several possible permutations of potential scenarios associated with the test results are described ( Table 1). Cows with a low P4 level were confirmed to be in oestrus if the cow was served and conceived or were confirmed not pregnant if the cow did not calve or was diagnosed as not pregnant. It was possible to identify cows that tested low but were pregnant by backward calculation from calving dates or PD foetal age estimates, however, it was not possible to confirm if any cows that had a low P4 result that did not conceive were not in oestrus. For cows with a high P4 level, pregnancy was confirmed by calving date, PD, or through an abortion or stillbirth event. For cows that had a high P4 level, it was possible to identify those that were not pregnant, however, it was not possible to confirm whether those cows had a high P4 level because they were not pregnant and also not in oestrus. Tests that returned a medium P4 level were considered inconclusive. 

**Table 1.** Possible scenarios associated with P4 Rapid test results.

P4 test result	Accurate	Inaccurate
**P4 level low**	Cow is in oestrus *or* Cow is not pregnant	Cow is not in oestrus * *or* Cow is pregnant
**P4 level medium**	Inconclusive	Inconclusive
**P4 level high**	Cow is not in oestrus * *or* Cow is pregnant	Cow is in oestrus *or* Cow is not pregnant

* Unable to determine with available information

Two scenarios, therefore, are presented; first a best-case scenario ( Table 2), where cows with a low P4 were actually in oestrus even though they did not conceive from the AI, and cows with a high P4 that were not pregnant were not in oestrus. Secondly, a worst-case scenario ( Table 3), is where all cows with a low P4 and did not conceive were counted as not being in oestrus and all cows with a high P4 that were not pregnant were counted as in oestrus. 

**Table 2.** P4 Rapid test accuracy – best-case scenario.

	Day 0	Day 21	Day 42	Overall
**Accurate**				
Test high and pregnant or not in oestrus	6	150	135	291
Test low and not pregnant	194	14	1	209
Total	200	164	136	500
Percentage %	97.1	96.5	98.6	97.3
**Inaccurate**				
Test high and not pregnant	0	0	0	0
Test low and pregnant	2	5	1	8
Total	2	5	1	8
Percentage %	1.0	2.9	0.7	1.6
**Inconclusive**				
Test medium and pregnant	3	1	1	5
Test medium and not pregnant	1	0	0	1
Total	4	1	1	6
Percentage %	1.9	0.6	0.7	1.2
**Total number**	206	170	138	514
**Total %**	100.0	100.0	100.0	100.0

**Table 3.** P4 Rapid test accuracy – worst-case scenario.

	Day 0	Day 21	Day 42	Overall
**Accurate**				
Test high and pregnant	6	132	123	261
Test low and not pregnant	176	11	1	188
Total	182	143	124	449
Percentage %	88.3	84.1	89.9	87.4
**Inaccurate**				
Test high and not pregnant or is in oestrus	0	18	12	30
Test low and pregnant or not in oestrus	20	8	1	29
Total	20	26	13	59
Percentage %	9.7	15.3	9.4	11.5
**Inconclusive**				
Test medium and pregnant	3	1	1	5
Test medium and not pregnant	1	0	0	1
Total	4	1	1	6
Percentage %	1.9	0.6	0.7	1.2
**Total number**	206	170	138	514
**Total %**	100.0	100.0	100.0	100.0

### Conception rate

Conception rates (cow conceived via a recorded AI service by the end of the study period) were compared between test and control groups ( Table 4). Cows that aborted or had a stillbirth were assumed to have conceived when last served.

**Table 4.** Conception rates for cattle.

	Test group	Control group	Total
**Conceived** **Did not conceive**	146 28	116 33	262 61
**Total** **Proportion conceived**	174 83.9%	149 77.9%	323 81.1%

Overall conception rates were higher in the test group (83.9%) compared to the control group (77.9%), although the difference was not significant (χ ^2^ = 1.9, p = 0.166).

### Abortion rate

Abortion rates (cows aborted via a recorded AI service by the end of the study period) were compared between test and control groups ( Table 5).

**Table 5.** Abortion rates for cattle.

	Test group	Control group	Total
**Aborted** **Did not abort**	12 134	11 105	23 239
**Total** **Proportion aborted**	146 8.2%	116 9.5%	262 8.8%

Overall abortion rates were higher in the control group (9.5%) compared to the test group (8.2%); however, the difference was not significant (χ ^2^ = 0.1, p = 0.720).

### Days to conception

**Figure 3.**
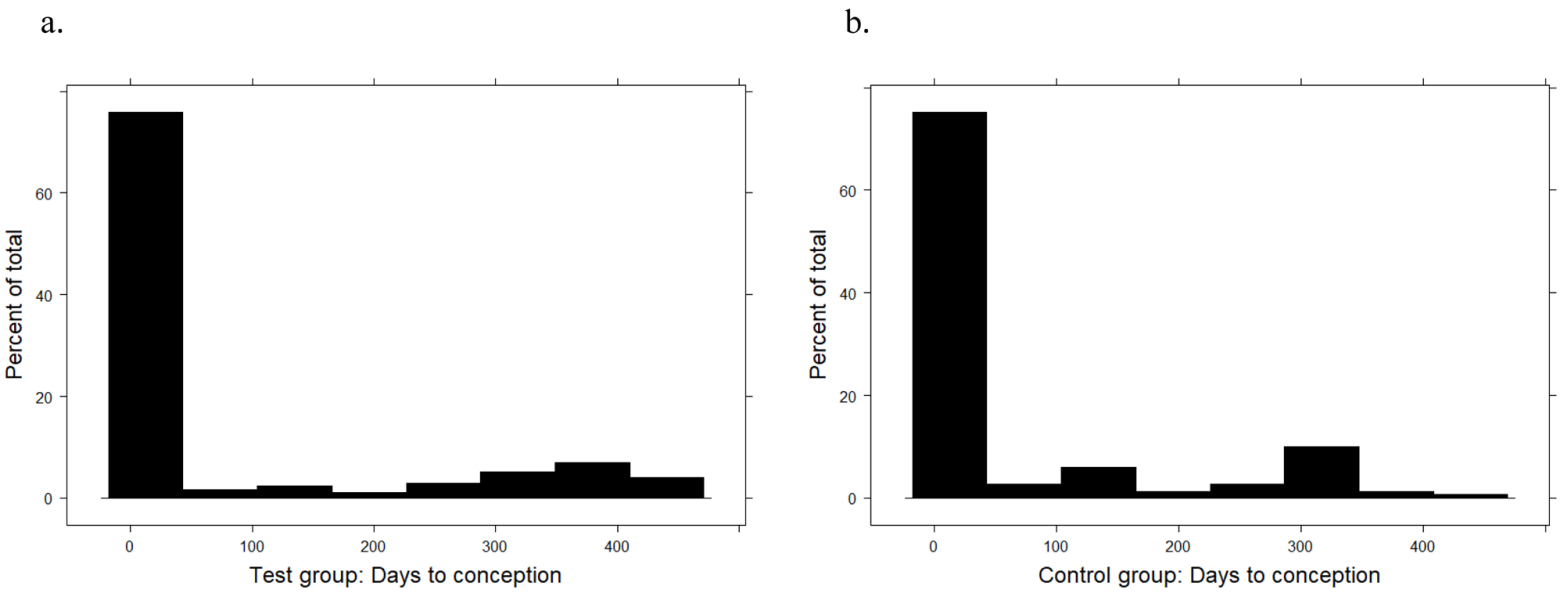
Summary statistic histograms of days to conception for a) test and b) control groups. Frequency (%) is on the y-axis and number of days on the x-axis.

The days to conception (DTC) were compared between test and control groups. Cows that aborted or had a stillbirth were included in the analysis, and considered to have conceived at their last recorded AI date. For cows that did not conceive during the study, DTC was calculated as the number of days between enrolment into the study (Day 0) and the study end date (1 December 2020). Summary statistics were calculated for test and control groups for DTC ( Table 6, Figure 3a and 3b).

**Table 6.** Summary statistics for days to conception (DTC) for test and control groups.

Group	Number of cattle	Mean	Median	Standard deviation	Interquartile range	Range
**Test**	174	76.04	0	142.1	0–21	0–453
**Control**	149	62.11	0	117.0	0–60	0–451

## Discussion

Both cultural and structural challenges constrain cattle breeding productivity in Kenya ( DeLay *et al.*, 2020). For example, whilst it has been demonstrated that the early detection of oestrus can reduce calving intervals, the preference for long lactations by some farmers can reduce or eliminate such advantages ( Odima *et al.*, 1994) and better record keeping could highlight the importance of fertility problems, abortions and tracking calving intervals ( Omore *et al.*, 1998). It is estimated that overall around 16% of smallholder farms in Kenya are using AI ( Mutavi *et al.*, 2016) although there are reports in some areas that uptake is as high as 94% ( Ajak *et al.*, 2020). In Nyandarua, high costs and inaccessibility of AI have been reported as prohibitive to around 60% of households, with smallholders choosing natural breeding methods instead ( Muia *et al.*, 2011), however there are other reports of higher uptakes ( Gitau, 2013). The timing of AI is essential to its success, which requires the accurate detection of oestrus, however, oestrus detection failure is a major constraint to reproductive performance ( Ball & Peters, 2004b). This has led to the development of technological aids to oestrus detection.

This study assessed the performance of the *P4 Rapid* progesterone lateral flow test in confirming oestrus and non-pregnancy in Kenyan dairy herds. A total of 745 tests were performed, of which 1.5% (11/745) were inconclusive (medium P4 level). In detecting oestrus, 93.8% (289/308) of all the cows in the test group presented for AI on day 0 had low P4 and were in oestrus. The study protocol required cows that were not in oestrus to be re-presented for AI the next time oestrus signs were visible, however, all repeat visits by farmers were on day 21 and day 42 regardless of day 0 test result. It is therefore not clear whether these cows were showing signs of oestrus on these days and so they were excluded from further calculations. Several possible scenarios associated with the test results were described and worst- and best-case scenarios presented. The true overall accuracy of the test performance likely lies somewhere between the extremes presented in these scenarios i.e. between 87–97%. A previous study, in Estonia, reported 98% accuracy in determining oestrus, and 84% accuracy in determining non-pregnancy, using the *P4 Rapid* test ( Waldmann & Raud, 2016).

Although there was no significant difference in pregnancy rates between the two groups, there was a tendency for higher rates in the test group (83.9%) compared to controls (77.9%). These pregnancy rates were much higher than anticipated, even in controls which may explain why there was no significant difference between tested and control cows. It has been noted previously that any beneficial response to a fertility management intervention is likely to be negatively proportionate to the background fertility of the herd before the intervention ( Peters, 1996, citing data from Drew & Peters, 1994) i.e. the poorer the fertility before intervention the greater the beneficial response to the intervention, and vice versa.

For cows with known outcomes, a total of 30 (10.3% of high test results) tests gave high P4 results in non-pregnant cows. It should be remembered that the *P4 Rapid* is not a pregnancy diagnostic tool. Conversely, there were eight instances where cows were pregnant but gave a low P4 test result (3.7% of low test results).

The study looked at the risk of abortion as a result of insemination of already-pregnant cows. In the test group, three cows were accidentally inseminated despite testing high; one cow was subsequently diagnosed as not pregnant so it is unclear if the cow aborted after AI or if the high P4 level was erroneous; one cow was confirmed as pregnant by ultrasound at 90 days and as the conception date was around the start of the study it was not clear if she was already pregnant, or became pregnant from AI; and one cow was lost to follow up i.e. unknown outcome. In the control group, 2.3% (5/219) of cows were already pregnant when served, based on subsequent calving date and backward calculation of conception date. However, for the cows that aborted, abortion date or foetal age were not always known and therefore it is not possible to determine whether the cow was already pregnant at AI – and AI was a causal factor – or whether the cow became pregnant from AI and then went on to abort. There are no recent published data on the risk of abortion by AI, and only very few older studies available. Vandemark *et al.* (1952) described 21 pregnant cows, in three groups; the seven cattle in group 1 were inseminated no further than the mid-point of the cervix and all had viable pregnancies; the eight cattle in group 2 were inseminated into the body of the uterus, with one cow aborting and seven resorbing foetuses; and the six cattle in group 3 were inseminated into the body of the uterus with antibiotics given, with four viable foetuses, one resorbing foetus and one partially destroyed foetal membranes but live foetus. Weaver *et al.* ( Weaver *et al.*, 1989) inseminated 57 oestrous cows; 25 were re-inseminated into the uterine body 12–24 days later while not in oestrus. Pregnancy rates were significantly lower in those re-inseminated (4%) compared to those not (40.6%). Sturman *et al.* ( Sturman *et al.*, 2000) observed embryo or foetal loss after insemination in 24% of pregnant cows, compared to 7% spontaneous loss of pregnancy otherwise occurring in the study. Moore *et al.* ( Moore *et al.*, 2005) reported the most important risk factor associated with embryo loss at 21–27 days and 28–35 days to be a second AI just prior or during these periods, with cattle 3.9 and 3.7 times, respectively, more likely to lose the embryo than those not re-inseminated. As well as the risk of iatrogenic abortion, mistiming of AI can also result in wasted semen and its associated expense.

There were obvious limitations in the study. The study was semi-experimental and not randomised, as this was thought to be potentially overly complicated for such a practical study setting on smallholder dairy farms. It was considered that too strict a protocol would reduce operator and farmer cooperation and compliance. This, therefore limited our ability to conclude causal association between the *P4 Rapid* test intervention and outcome(s). Although the study was planned as a direct comparison of fertility parameters between tested cows and non-tested (control) cows, no significant difference in pregnancy rates was found and thought to be due to the relatively good background fertility management in the selected herds. This suggests that the efficiency of oestrus detection was quite good in these herds and therefore the incremental benefit of confirmation by P4 testing could only be expected to be marginal. In this situation, therefore, routine testing is clearly not warranted on economic grounds but selection of cows for P4 testing should be based on doubtful or problem cases. Further work is planned on the potential economic impact of cow-side progesterone testing in sub-Saharan Africa.

In conclusion, the *P4 Rapid* test is considered a useful tool to help Kenyan dairy farmers overcome the key challenges in the timing of AI, particularly where the level of oestrus detection is poor. Whilst its accuracy is imperfect, it compares favourably to quantitative progesterone assays but is more suitable and accessible for the smallholder setting, as a cow-side test, without the necessity for refrigeration or laboratory facilities. Additionally, and importantly, the *P4 Rapid* test may assist farmers in reducing AI-induced abortions by avoiding the insemination of pregnant cattle.

## Data Availability

Harvard Dataverse: Use of a cow-side oestrus detection test for fertility management in Kenyan smallholder dairy herds: Supporting Data. https://doi.org/10.7910/DVN/OOVYY1 ( Allan, 2022). This project contains the following underlying data: Kenya P4 Rapid cleaned database.xlsx Harvard Dataverse: Use of a cow-side oestrus detection test for fertility management in Kenyan smallholder dairy herds: Supporting Data. https://doi.org/10.7910/DVN/OOVYY1 ( Allan, 2022). This project contains the following extended data: AI Training course.pdf Data are available under the terms of the Creative Commons Zero "No rights reserved" data waiver (CC0 1.0 Public domain dedication).
