# Supplementary material for: Use of a cow-side oestrus detection test for fertility management in Kenyan smallholder dairy herds
Source: Gates Open Res. 2022 Sep 20;6:12. Originally published 2022 Mar 14. [Version 2] doi: 10.12688/gatesopenres.13542.2 (PMC9352916; doi:10.12688/gatesopenres.13542.2)
Supplement: Peer review history

# Peer review: Reviewer response for version 1

**DOI:** 10.21956/gatesopenres.14807.r32238

**Published:** 2022-08-04

**Authors:** Leigh Olufisayo Oluwadamilare

**Affiliations:** 1 Department of Theriogenology, Faculty of Veterinary Medicine, University of Ibadan, Ibadan, Nigeria

The criteria used to decide animals to inseminate in the test group were not so precise: low, medium and high P4. However, the finding that 6.2 percent of those who initially showed high P4 were eventually pregnant suggests a substantial level of reliability for the cow-side P4 test.

  

- The methodology raises concern on the level of the precision of farmers and the P4 test. For the test group, the authors claimed that animals were presented, just as in the control group, when farmers saw oestrus signs. Why, then, did the P4 test return such animals as either inconclusive or high?
- On pregnancy diagnosis, the text suggests that calving information was obtainable between days 60 and 90 post-insemination. Please clarify.
- Data analysis is ok. On Table 1, please change 'Equivocal' to 'Inconclusive', as contained in other parts of the manuscript.
- In the third paragraph of 'Discussion', what were the anticipated pregnancy rates for test and control cows? And on what basis would authors fix an outcome before concluding the study? Was this related to what previous authors have reported while using the same P4 rapid test kit?
- The study is novel, no doubt. Not only in terms of considerable precision regarding animals to be inseminated, but also in identifying animals at risk of induced abortion due to wrongful insemination. However, some areas remained unclear in the interpretation of the study outcome.
- Although the proportion of cows that were pregnant (by calving date calculation) and inseminated wrongfully was low, the comparative advantage of the P4 rapid test is brought to question if one considers the socio-economic value of the eventual loss of such pregnancies. Though this occurs naturally in the traditional oestrus detection methods leading to insemination, it is not known if the proportion of wrongful insemination in the traditional method is lower or surpasses that recorded with the P4 rapid test.

By and large, this is a well-researched and concluded study.

Is the work clearly and accurately presented and does it cite the current literature?

Yes

If applicable, is the statistical analysis and its interpretation appropriate?

Yes

Are all the source data underlying the results available to ensure full reproducibility?

No source data required

Is the study design appropriate and is the work technically sound?

Yes

Are the conclusions drawn adequately supported by the results?

Yes

Are sufficient details of methods and analysis provided to allow replication by others?

Yes

Reviewer Expertise:

Assisted Reproduction Techniques, Veterinary Obstetrics

I confirm that I have read this submission and believe that I have an appropriate level of expertise to confirm that it is of an acceptable scientific standard.

# Response

**Published:** 2022-08-15

**Authors:** Fiona Allan

**Affiliations:** Royal (Dick) School of Veterinary Studies, UK

Dear Reveiwer, 

 We sincerely thank you for your time and effort in providing expert assessment of our manuscript. There are constructive points raised which we have considered and hope that we have addressed suitably.

 Responses to comments are listed in italic font below, with additions and amendments highlighted in bold.

 1.  The methodology raises concern on the level of the precision of farmers and the P4 test. For the test group, the authors claimed that animals were presented, just as in the control group, when farmers saw oestrus signs. Why, then, did the P4 test return such animals as either inconclusive or high?

*The test was used to confirm oestrus or otherwise. In some cases the observation may be mistaken. In these circumstances the test result prevents a mis-timed insemination e.g. the cow may already be pregnant and repeat AI could cause abortion.  We thought we had explained this in the paper page 5.*

 2.  On pregnancy diagnosis, the text suggests that calving information was obtainable between days 60 and 90 post-insemination. Please clarify.

*Pregnancy was confirmed by 60-90 day ultrasound PD and/or by subsequent calving date.*

 3.  Data analysis is ok. On Table 1, please change 'Equivocal' to 'Inconclusive', as contained in other parts of the manuscript.

***We will amend this in the manuscript.***

 4.  In the third paragraph of 'Discussion', what were the anticipated pregnancy rates for test and control cows? And on what basis would authors fix an outcome before concluding the study? Was this related to what previous authors have reported while using the same P4 rapid test kit?

*There was anecdotal evidence (there is a dearth of published literature on bovine pregnancy rates in Kenya) that pregnancy rates would be much lower, as it has been one of the main reasons cited for lack of uptake of cattle AI in sub-Saharan Africa.  It was not related to previous use of the P4 kit.  We had expected control cow pregnancy rates to be around 50%. It is inferred that the herds selected were experienced in using AI and thus in efficient oestrus detection.*

 5.  The study is novel, no doubt. Not only in terms of considerable precision regarding animals to be inseminated, but also in identifying animals at risk of induced abortion due to wrongful insemination. However, some areas remained unclear in the interpretation of the study outcome.

*We have looked again at our description of the Methods, Results and Discussion and feel that we have articulated the study as clearly as we can.*

 6.  Although the proportion of cows that were pregnant (by calving date calculation) and inseminated wrongfully was low, the comparative advantage of the P4 rapid test is brought to question if one considers the socio-economic value of the eventual loss of such pregnancies.

*We believe that the socio-economic consequences of identifying already-pregnant cows increase the advantage of use of P4.*

 7.  Though this occurs naturally in the traditional oestrus detection methods leading to insemination, it is not known if the proportion of wrongful insemination in the traditional method is lower or surpasses that recorded with the P4 rapid test.

*We believe that for first inseminations, when P4 confirms ovulation after an observed oestrus, the proportion of mis-timed inseminations can only be equal or lower.*

 By and large, this is a well-researched and concluded study. 

*Thank you. This is much appreciated.*

# Peer review: Reviewer response for version 1

**DOI:** 10.21956/gatesopenres.14807.r31868

**Published:** 2022-04-19

**Authors:** Alemayehu Lemma

**Affiliations:** 1 College of Veterinary Medicine and Agriculture, Addis Ababa University, Bishoftu, Ethiopia

**Abstract:**

- The discouraging matter towards using AI is just its poor success. In most cases cost of the service is either relatively cheap or subsidized by governments. If this is only true in Kenya, it should then be specifically mentioned so.

**Methods:**

- The categorization of P4 level as low, medium and high must be referenced to a specific concentration of P4. We know P4 level below 1ng is considered showing accurately either an oestrous or non pregnancy.
- More discrete description of selection criteria would be better instead of generalized description as “Body condition score and health information were recorded for each cow, including cases of mastitis and other infections” e.g. BCS, parity, breed type, health etc. can influence the outcome of the study.
- The statement “Oestrus was determined by the farmer by observing behavioural signs and oestrous cycle tracking as described by Ball and Peters” - it is not clear whether the farmers were previously trained on this method. Please indicate how farmers got the skill/knowledge to detect oestrus as described by Ball and Peters.
- Pregnancy Diagnosis should have been performed by a more accurate method (ultrasonography) as early as possible (21 days post AI). Failure to do so and base results obtained from later stage can undermine the performance of the P4 Rapid test because there could be early embryonic losses up to day 60.

Is the work clearly and accurately presented and does it cite the current literature?

Yes

If applicable, is the statistical analysis and its interpretation appropriate?

Yes

Are all the source data underlying the results available to ensure full reproducibility?

Yes

Is the study design appropriate and is the work technically sound?

Yes

Are the conclusions drawn adequately supported by the results?

Yes

Are sufficient details of methods and analysis provided to allow replication by others?

Partly

Reviewer Expertise:

Reproduction/Reproductive biotechnology

I confirm that I have read this submission and believe that I have an appropriate level of expertise to confirm that it is of an acceptable scientific standard.

# Response

**Published:** 2022-04-26

**Authors:** Fiona Allan

**Affiliations:** Royal (Dick) School of Veterinary Studies, UK

Dear Reviewer,

 We sincerely thank you for your time and effort in providing expert assessment of our manuscript. There are constructive points raised which we have considered and hope that we have addressed suitably.

 Response to comments are listed in italic font below, with amendments in bold.

**Abstract:**

 The discouraging matter towards using AI is just its poor success. In most cases cost of the service is either relatively cheap or subsidized by governments. If this is only true in Kenya, it should then be specifically mentioned so.

*We would agree with this comment, a lot of the problems with AI is poor fertility rather than oestrus detection; we do mention this, with citations saying that oestrus detection is a problem.*

*The low fertility rates will likely be due to poor nutrition both in terms of energy and protein but also mineral status with little testing for the important minerals for fertility being carried out (Copper/Selenium/Cobalt/Phosphorous and Iodine).*

***We can rephrase as ‘poor success and, in some situations, high costs in Kenya, have been discouraging’.***

**Methods:**

 The categorization of P4 level as low, medium and high must be referenced to a specific concentration of P4. We know P4 level below 1ng is considered showing accurately either an oestrous or non pregnancy.

***We can add that medium is > 2 ng/ml and ≤ 3.5 ng/ml, and high is > 3.5 ng/ml (Roelofs et al., 2006)***

 More discrete description of selection criteria would be better instead of generalized description as “Body condition score and health information were recorded for each cow, including cases of mastitis and other infections” e.g. BCS, parity, breed type, health etc. can influence the outcome of the study.

*Rather than adding more discrete health conditions, **we could add that cows were excluded if they were suffering from a health issue which could affect fertility**.*

 The statement “Oestrus was determined by the farmer by observing behavioural signs and oestrous cycle tracking as described by Ball and Peters” - it is not clear whether the farmers were previously trained on this method. Please indicate how farmers got the skill/knowledge to detect oestrus as described by Ball and Peters.

***Farmers were already using AI and so were using AI providers, and oestrus detection was according to normal, widely and internationally recognized farm practice, as described by Ball and Peters.  In Kenya, it is common practice that genetic supply companies, in collaboration with AI service providers, routinely offer embedded extension and farmer education on factors affecting reproduction efficiency, including the equation of reproduction that includes four key factors: 1) efficiency of the AISP, 2) efficiency of heat detection, 3) cow fertility, and d) bull semen fertility.***

 Pregnancy Diagnosis should have been performed by a more accurate method (ultrasonography) as early as possible (21 days post AI). Failure to do so and base results obtained from later stage can undermine the performance of the P4 Rapid test because there could be early embryonic losses up to day 60.

***This is a valid point and should be added into the protocol. ** Due to constraints of working with smallholder farmers in limited resource settings, it was not possible to ultrasound PD all of the cows. Additionally, scanning at 21 days would not be accurate, adding little to the P4 results in terms of estimating incidence of early fetal death.*
